# Size extensivity of elastic properties of alkane fragments

**DOI:** 10.1007/s00894-017-3572-9

**Published:** 2018-01-08

**Authors:** Milad Radiom, Plinio Maroni, Tomasz A. Wesolowski

**Affiliations:** 1 Department of Inorganic and Analytical Chemistry, University of Geneva, Quai Ernest-Ansermet 30, 1205 Geneva, Switzerland; 2 Present Address: School of Chemical Science and Engineering, KTH Royal Institute of Technology, Drottning Kristinas väg 51, 10044 Stockholm, Sweden; 3 Department of Physical Chemistry, University of Geneva, Quai Ernest-Ansermet 30, 1205 Geneva, Switzerland

**Keywords:** Elasticity constant, Quantum chemistry, Atomic force microscopy, Single molecule extension response

## Abstract

Using MP2, CCSD, and B3LYP methods of computational chemistry, we show length dependence in the intrinsic elastic properties of short alkane fragments. For isolated alkane fragments of finite length in the gas phase and zero temperature, the intrinsic elasticity constants are found to vary with the number of carbon atoms and its parity. From extrapolation of the elasticity constants calculations to infinite chain length, and by comparing with in-situ elasticity constant of single poly(ethylene) molecule obtained with atomic force microscopy, we estimate the softening effect of environment on the extension response of the polymer.

**Electronic supplementary material:**

The online version of this article (10.1007/s00894-017-3572-9) contains supplementary material, which is available to authorized users.

## Introduction

Quantum chemistry (QC) is an established tool to calculate molecular structure and energy. Application of its methods is recently extended to the investigation of polymer molecules extension response using atomic force microscopy (AFM) [1–7], but is also applicable to other force microscopy techniques [8–10]. In the QC approach, the energy profile of a short polymer fragment is computed as a function of a constrained geometry, which is normally the end-to-end extension. Usually derivatives of this energy profile are used to calculate the elastic properties of the fragment, which are then used in interpretation of in-situ elastic response of polymer molecules [11–13]. Within these efforts, conformational transition of poly(ethylene oxide) was modeled using the QC energy profile [2, 7, 13], and extension response of various polymers were modeled using the QC elasticity constants [3–6, 12].

Significant differences exist between QC fragments and the actual polymer molecules. For example, polymer molecules are at least two orders of magnitude longer than the fragments used in the computations. Experiments are performed in solvent environment while computations at gas phase and zero temperature. To circumvent these differences, it is assumed that the computed elasticity constants are intrinsic properties of the short fragments. It is also usually assumed that the gas-phase zero-temperature elasticity constants are preserved in situ.

Previous work has supported some of these assumptions. For example it was shown that the energy profiles of propane, heptane, and undecane when normalized by their lengths agree reasonably, and thereby their elasticity constants are intrinsic properties of these fragments [11]. These computations were performed using Hartree-Fock (HF) method which is a low level of computational theory due to its exclusion of electron-electron interaction correlations. The previous work also investigated an extension range with tensile force up to about 12 nN that is beyond accessible to current AFM single molecule force microscopy methods with an upper limit of about 2 nN [3–6]. We have reproduced these results using a similar method and found agreement with previous work over a similarly long extension range, see Fig. S1. However, investigation of the energy profiles in AFM-related range (up to about 2 nN), using higher levels of computational theory, shows that the normalized energy profiles of alkane fragments do not actually overlap. While length dependence in elasticity constant of DNA molecules computed using molecular dynamics (MD) simulation has been reported [14], we are not aware of any QC method with inclusion of correlation energy that have been used to accurately examine the elasticity constants of alkane fragments and their size dependence.

The impact of experimental condition, as compared with gas-phase zero-temperature condition of computations on molecular processes was addressed by Politzer and co-workers [15]. These authors showed computationally that solvent may facilitate structural changes of molecules in a chemical process. In particular, forces near the equilibrium geometry are weakened in the presence of the solvent (see Fig. 2b in ref. [15]). The elastic properties of DNA molecules showed variations with salt concentrations of monovalent and divalent salts using MD simulations [14, 16]. The effect of temperature was addressed in MD simulations of poly(ethylene glycol) monomers [17]. In the force-extension profiles, the structural transition observed at 250 K disappears at higher simulation temperatures where the force-extension profiles do not show significant variations (see Fig. S9a in ref. [17]).

We show that the computed elasticity constants of short alkane fragments are not intrinsic properties of these fragments. The elasticity constants vary with the length of the fragment or the number of carbon atoms and its parity. We extrapolate the computed elasticity constants to infinite chain length and compare with the elasticity constant of poly(ethylene) obtained with AFM-based single molecule force microscopy. This comparison gives an estimation of the contribution of the environment on the elastic response of the polymer.

## Methods and materials

The computations are performed for fragments of the simplest polymer that is poly(ethylene). We employ Moller-Plesset-2 perturbation (MP2) method which adds second order corrections to HF energy from electron pair correlations from doubly excited states [18]. We use four types of split valence basis sets including polarization functions. In Pople style, these are 6-31G*, 6-31G**, 6-311G*, and 6-311G** [19, 20]. The computations are performed for alkane fragments of various lengths starting from the shortest, propane, to the longest, dodecane. Dodecane is the longest fragment that we study as its contour length is slightly shorter than the length expected to induce bent structures [21, 22].

Our MP2 conclusions are further verified using coupled cluster with singly and doubly excitations (CCSD) method which adds infinite order corrections to HF energy from electron correlations of singly and doubly excited states and is more accurate than MP2 [23]. We perform additional computations using density functional theory (DFT) using B3LYP exchange-correlation functional [24, 25]. With CCSD and B3LYP methods, 6-31G* basis set is used. Computations using MP2 method and B3LYP method are performed for all fragments. To reduce computational costs, simulations using CCSD method are performed only for propane, butane, heptane, octane, undecane, and dodecane. All computations are performed using Gaussian 09 program [26].

We start with geometry optimization in the ground state where energy of a fragment is minimized by optimizing all its degrees of freedom including bond lengths, bond angles, and dihedral angles. This procedure results in the ground state energy *E*_0_ and the contour length *L* which is the distance between terminal carbon atoms in the fragment in the ground state.

The energy-extension profile is obtained in a procedure which consists of varying the distance *x* between terminal carbon atoms from the ground state. This distance is constrained while all the remaining degrees of freedom are optimized to obtain a minimized energy.

To obtain the elasticity constants the energy-extension profile is fitted to a polynomial [4]

1 $$ E=L\cdot \sum \limits_{n=2}{K}_{n-1}{\left(\frac{x}{L}-1\right)}^n/n $$

where *K*_n ‐ 1_ is the elasticity constant, and *E* is the energy relative to the ground state. From the derivative of the energy versus extension profile one obtains the force

2 $$ F=\sum \limits_{n=2}{K}_{n-1}{\left(\frac{x}{L}-1\right)}^{n-1} $$

which is the parameter measured in AFM single molecule measurements [3–6].

Details of the AFM experiments are explained elsewhere [27] and data reprocessed here. Poly(ethylene) was purchased from Sigma Aldrich and had a molecular mass of 4500 kg mol^−1^. A small piece of naturally oxidized silicon wafer (Silchem, Germany) was cleaned for 20 min with piranha solution, which consisted of a mixture of H_2_SO_4_ 98% and H_2_O_2_ 30% in a volumetric ratio of 3:1. The piece was then rinsed with Milli-Q water and dried in a stream of nitrogen. It was then treated with oxygen-enriched UV-ozone cleaner (PSD Pro, Novascan, Ames, USA) for 20 min. The clean piece was then immediately used for polymer deposition. The substrate was coated with the polymer by placing a 10 μL drop of polymer solution of a concentration of 100 mg/L in toluene for 20 min on its top. The coated substrate was then rinsed with toluene, dried in a stream of nitrogen, and mounted in the fluid cell of the AFM (Cypher, Oxford Instruments). The interaction between an AFM-tip (BL-AC40TS, Olympus, Japan, spring constant 0.1 N/m measured by thermal method [28]) and this substrate was probed with repeated approach-retraction cycles with a velocity of 200 nm/s. Force–distance profiles were acquired with a sampling rate of 2 kHz, and were subsequently converted to force–extension profiles by subtraction of the tip deflection [29].

The stretching part of the force profile is analyzed by least squares fit with the modified freely jointed chain (FJC) model which contains elasticity constant. This model predicts that the applied force *F* is related to the extension *x* of the chain by [13, 27, 30]

3 $$ \frac{x}{L}=\coth \left(\frac{\mathrm{\ell}F}{kT}\right)-\frac{kT}{\mathrm{\ell}F}+\frac{F}{K} $$

where ℓ is the apparent Kuhn length, *K* the elasticity constant, *k* the Boltzmann constant, and *T* the absolute temperature. In FJC model the elasticity constant term is approximated by Hooke’s law. Considering the accuracy of AFM force measurements, which is limited by errors due to determination of the spring constant of AFM tip and thermal noise, we suspect that quadratic approximation to the elastic response of polymer will remain valid in the extension range examined by the AFM, up to about 2 nN.

## Results and discussion

**Fig. 1:**
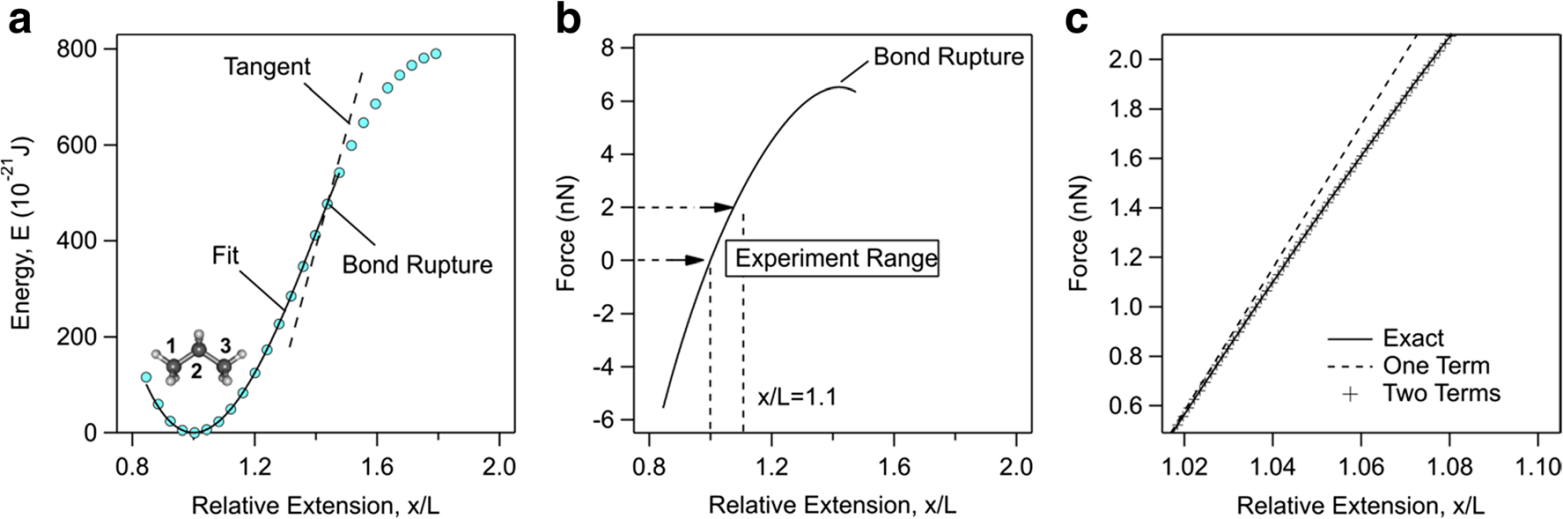
(a) Energy and (b) force as a function of relative extension of propane together with the best fit of Eq. (1) to the energy profile using three elasticity constant terms. Bond rupture corresponds to inflection point on energy profile and maximum on force profile. Computations are performed using MP2/6-31G* method and the resulting elasticity constant terms are *K*_1_ = 29.0 nN, *K*_2_ = − 37.9 nN, and *K*_3_ = 38.6 nN. Experimentally, forces of up to about *F* = 2 nN are obtained with AFM which corresponds to relative extension *x*/*L* = 1.1. The experimental range is shown on (b). (c) Comparison between complete force profile, and approximate profiles using one elasticity constant term and two elasticity constant terms in the experimental range

Typical computations of energy-extension profile with MP2/6-31G* method for propane are shown in Fig. 1a. The terminal carbon atoms, C1 and C3, are extended from an initially contracted molecule to an extended one. The ground state corresponds to minimum of the energy profile. At relative extension *x*/*L =* 1.4, the inflection point corresponds to carbon-carbon bond rupture.

We fit the energy-extension profile of propane in Fig. 1a with Eq. (1) and obtain *K*_1_ = 29.0 nN, *K*_2_ = − 37.9 nN, and *K*_3_ = 38.6 nN. These elasticity constants are in close agreement with computations of Hugel et al. [4]. These authors found *K*_1_ = 28.7 nN, *K*_2_ = −42.0 nN and *K*_3_ = 16.9 nN using MP2 method and triple zeta valence (TZV) basis set.

The force-extension profile of propane is shown in Fig. 1b. One observes that the inflection point on the energy profile (Fig. 1a) corresponds to maximum in force profile with value 6 nN. This rupture force is however much larger than that is accessible to AFM single molecule force microscopy experiments where a force of about 2 nN is normally obtained [3–6]. As shown in Fig. 1b force *F* = 2 nN corresponds to relative extension *x*/*L* = 1.1. To comply with AFM experiments, we limit our subsequent energy computations to relative extensions *x*/*L* = 1 to 1.1. Figure 1c shows that, within this range, a force profile calculated from only the first two elasticity constant terms, the quadratic and cubic terms, gives an excellent agreement with the complete force profile. Using only one elasticity constant term results in 10% deviations from the complete profile at large forces. This error is small when compared with uncertainties in AFM force measurements. Thereby, the quadratic approximation provides enough accuracy when applied to AFM results. This result shows that up to a force of about 2 nN using only the quadratic elasticity constant term still provides an adequate accuracy in the force. It is worthwhile to recall here the studies of the elastic properties of carbon nanotubes by Politzer and co-workers [31]. In the linear polymers investigated in the present work and in the carbon nanotubes investigated in ref. [31], the nature of the carbon-carbon bonds is significantly affected at maximally stretched molecule (relative extension of the overall length of the molecule equal to 10%). Despite this fact, the overall shape of the energy profile is dominated by the quadratic term in either case. Nevertheless, we fit the rest of the energy-extension profiles in this work using two elasticity constant terms.

**Fig. 2:**
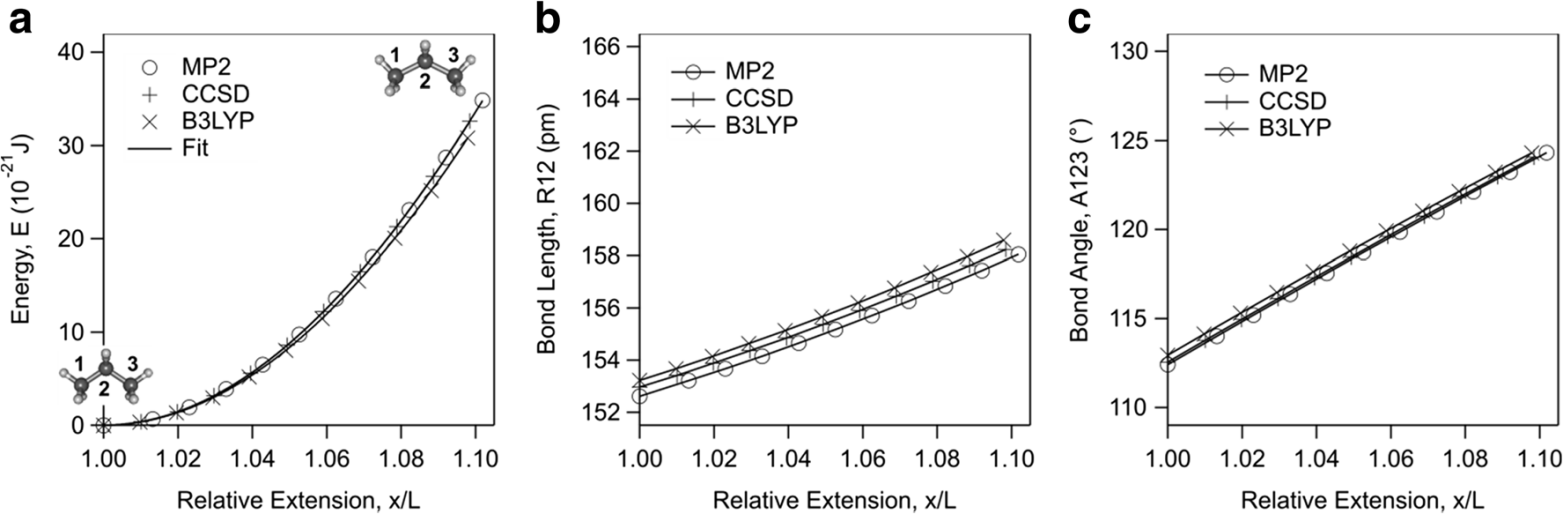
(a) Energy, (b) bond length, and (c) bond angle as a function of relative extension of propane together with the best fit of Eq. (1) to energy profile using two elasticity constant terms. The resulting elasticity constants are *K*_1_ = 29.0 nN and *K*_2_ = −37.9 nN with MP2/6-31G* method, *K*_1_ = 29.0 nN and *K*_2_ = −38.7 nN with CCSD/6-31G* method, and *K*_1_ = 27.3 nN and *K*_2_ = −33.0 nN with B3LYP/6-31G* method. Bond length R12 is between carbon atoms 1 and 2. Bond angle A123 is between carbon atoms 1, 2, and 3

The energy-extension profile of propane is computed with CCSD and B3LYP theories using 6-31G* basis set and compared with MP2 computations in Fig. 2a. We observe insignificant differences between the computations of these theories. The difference between the MP2 profile and the CCSD profile is negligible showing that the computations with MP2 are converged. Fitting with Eq. (1) gives *K*_1_ = 29.0 nN and *K*_2_ = −38.7 nN with CCSD method and *K*_1_ = 27.3 nN and *K*_2_ = −33.0 nN with B3LYP method, again in close agreement with MP2 calculations.

For each method, the variations of the bond length and bond angle are computed and compared in Fig. 2b and c. Once again very good agreement between the theories is observed. One observes that the bond length R12 between carbon atoms 1 and 2, and the bond angle A123 between carbons atoms 1, 2, and 3 increase monotonically with the end-to-end extension.

**Fig. 3:**
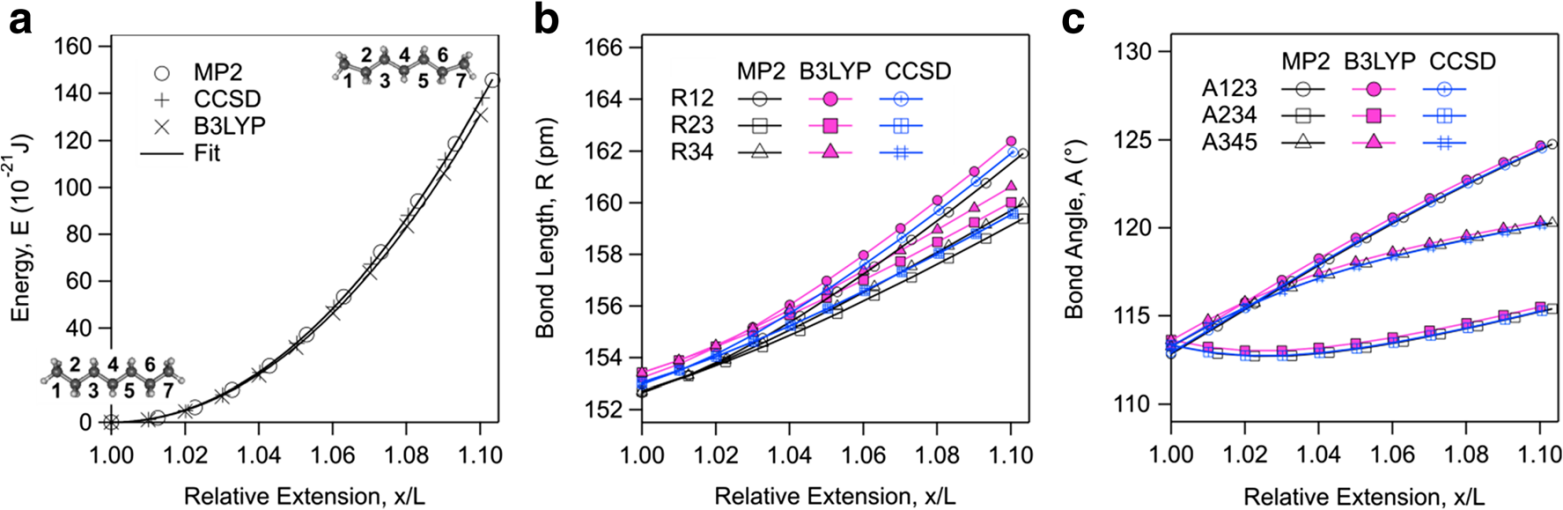
(a) Energy, (b) bond length, and (c) bond angle as a function of relative extension of heptane together with the best fit of Eq. (1) to energy profile using two elasticity constant terms. The resulting elasticity constants are *K*_1_ = 34.1 nN and *K*_2_ = 24.5 nN with MP2/6-31G* method, *K*_1_ = 34.1 nN and *K*_2_ = 24.7 nN with CCSD/6-31G* method, and *K*_1_ = 32.5 nN and *K*_2_ = 23.3 nN with B3LYP/6-31G* method. Bond lengths R12, R23, and R34 are between carbon atoms 1 and 2, 2 and 3, and 3 and 4 respectively. Bond angles A123, A234, and A345 are between carbon atoms 1, 2 and 3, 2, 3 and 4 and 3, 4 and 5

We run similar computations with longer alkane fragments from butane to dodecane. Figure 3 shows the energy, bond length, and bond angle profiles of heptane as a function of relative extension between terminal carbon atoms C1 and C7 computed with MP2/6-31G* method. Fitting the energy-extension profile with Eq. (1) using the quadratic and cubic elasticity constant terms gives *K*_1_ = 34.1 nN and *K*_2_ = 24.5 nN (Fig. 3a). In Fig. 3b it is shown that the variations of the bond lengths R12, R23, and R34, respectively between carbon atoms 1 and 2, 2 and 3, and 3 and 4 are only marginally different. At relative extension *x*/*L* = 1.1, we find R12 = 0.164 nm, R23 = 0.161 nm, and R34 = 0.162 nm. Figure 3c shows, however, that the variations in the bond angles A123, A234 and A345, respectively between carbon atoms 1, 2 and 3, 2, 3 and 4, and 3, 4 and 5, are very different. One observes that A123 at the end of the fragment varies the most while the smallest variation is observed for A234 which is the immediate next bond angle. The computations are repeated with CCSD/6-31G* and B3LYP/6-31G* theories and give excellent agreement with MP2 method. Fitting the energy-extension profile with Eq. (1) gives *K*_1_ = 34.1 nN and *K*_2_ = 24.7 nN with CCSD method and *K*_1_ = 32.5 nN and *K*_2_ = 23.3 nN with B3LYP method. The close agreement between MP2 and CCSD theories again shows convergence of the MP2 computations.

One observes that while variations of the bond lengths in heptane and in propane are somewhat similar, the variations in the bond angles are very different. Only the bond angle at the end of heptane, A123, varies similarly to the bond angle in propane, A123. The inner bond angles in heptane are found to vary to a much lesser extent. Since within a similar relative extension, variations of the inner bond angles in heptane are smaller, they result in a relatively stiffer extension response as compared with propane. This relatively stiffer response is evident from comparison between their elasticity constant terms.

**Fig. 4:**
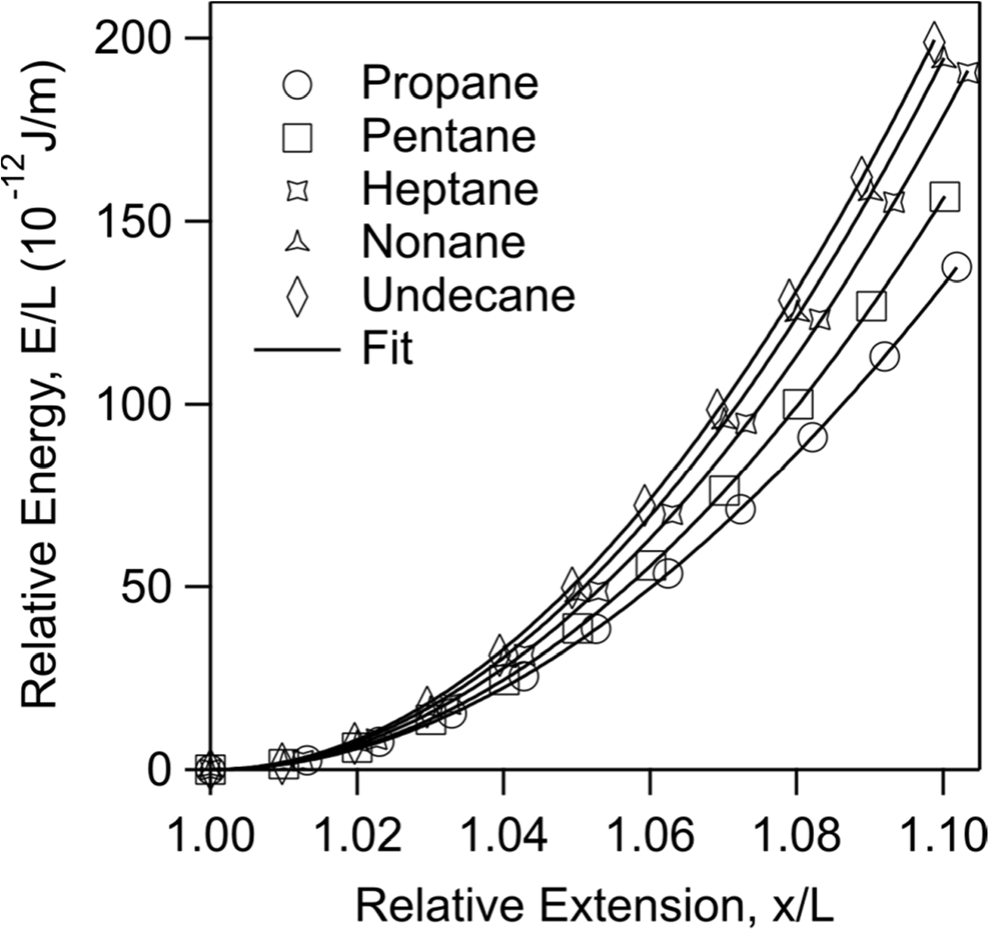
Relative energy as a function of relative extension of alkane fragments with odd number of carbon atoms together with the best fit of Eq. (1) using two elasticity constant terms. Computations are performed with MP2/6-31G* method

The form of Eq. (1) suggests that a plot of relative energy (*E* divided by *L*) as a function of relative extension should overlap. Figure 4 shows this plot for propane, pentane, heptane, nonane, and undecane computed with MP2/6-31G* method. One observes that the relative energy profiles of these fragments do not overlap in the entire relative extension range. This is contrary to previous computations of similar fragments using HF method [11]. Figure 4 shows that the relative energy increases with the size of the fragments. This result clearly shows that the energy-extension profiles of short alkane fragments depend on their length. The difference between the relative energy profiles, however, diminishes with the length of the fragments.

**Fig. 5:**
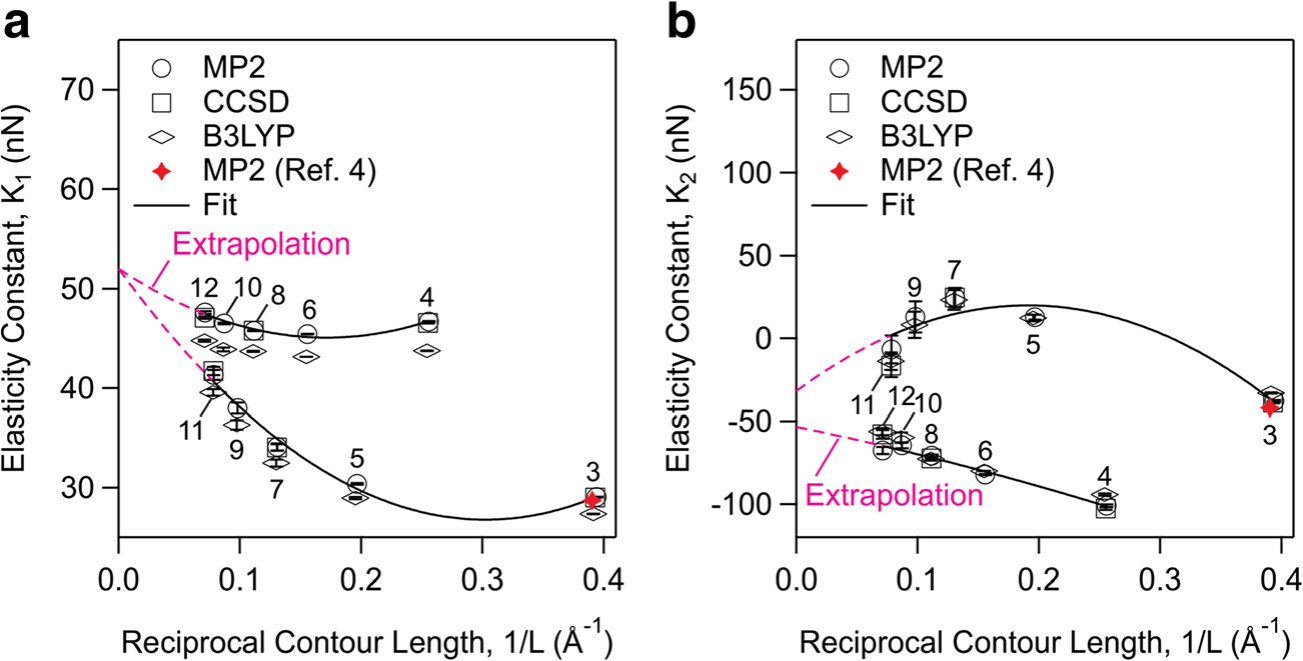
The quadratic (a) and cubic (b) elasticity constant terms from MP2, CCSD, and B3LYP methods plotted as a function of reciprocal contour length. The elasticity constant terms alternate between even or odd number of carbon atoms, these numbers are shown on each figure. The elasticity constant terms from MP2 method are fit to Eq. (4) where the same fit is extrapolated to infinite chain length. The resulting fit parameters are given in Table S2. Extrapolation to infinite chain is shown with broken line. The extrapolation gives *K*_1_ = 51.9 ± 0.3 nN and *K*_2_ = −42.6 ± 10.7 nN for infinite chain length in gas phase and zero temperature. Error bars are 95% confidence interval in the fitted parameters. MP2 computations from ref. [4] with propane are added for comparison

The quadratic and cubic elasticity constant terms obtained from fits of Eq. (1) to the computed energy-extension profiles of all the fragments are plotted as a function of reciprocal contour length in Fig. 5. One observes alternations in the elasticity constant values with respect to odd or even number of carbon atoms. We suspect that this alternation is due to the difference in the point symmetries of the fragments. An even number of carbon atoms forms a C(2 h) point symmetry while an odd number a C(2v) symmetry. Different symmetries may result in different distribution of forces among the atoms. The alternations are nevertheless observed to diminish with increasing contour length which provides an opportunity for extrapolation of the elasticity constant terms to infinite chain length. Increasing the size of basis set to 6-31G**, 6-311G*, and 6-311G** is shown to have no effect on these results (Fig. S2).

To estimate the quadratic and cubic elasticity constant terms for an infinite chain length we fit the elasticity constant terms with relation

4 $$ K=a+\frac{b}{L}+\frac{c}{L^2} $$

where *a*, *b*, and *c* are constants. The fits are shown in Fig. 5, while the values of the best fit constants are given in Table S2. The fits are then extrapolated to infinite chain length, 1/*L* → 0. The extrapolation gives *K*_1_ = 51.9 ± 0.3 nN and *K*_2_ = −42.6 ± 10.7 nN for an infinite chain length.

**Fig. 6:**
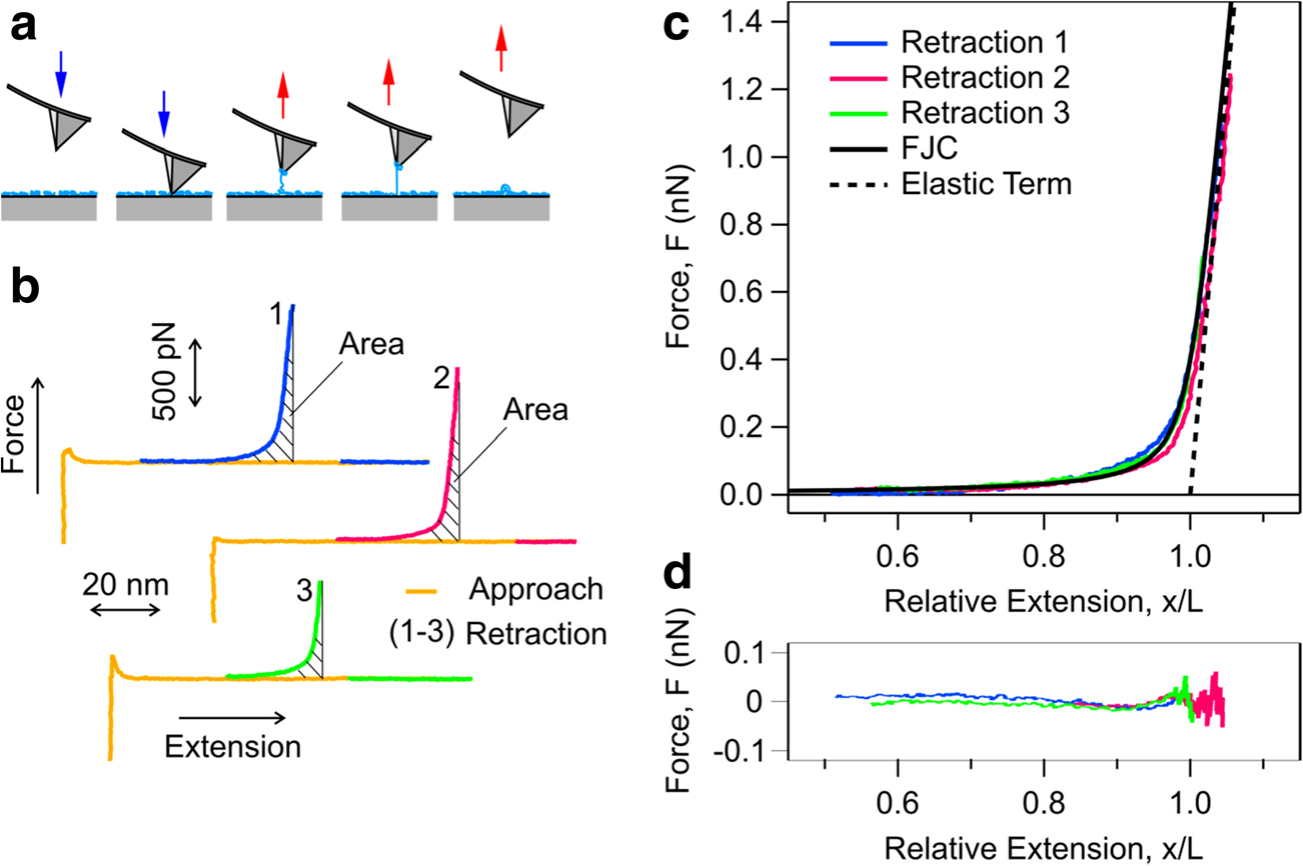
Probing the extension response of single poly(ethylene) polymer molecules by AFM. (a) Scheme of the experiment. (b) Representative approach and retraction force–extension profiles. The shaded area under force–extension profile denotes work applied by AFM cantilever. (c) Force profiles plotted as a function of relative extension together with the best fit with FJC model. The resulting fitting parameters are Kuhn length ℓ= 0.6 ± 0.1 nm and elasticity constant *K* = 24 ± 3 nN. The residuals are shown in (d)

At this point it is interesting to compare the extrapolated elasticity constant terms with elasticity constant of poly(ethylene) obtained with AFM single molecule force microscopy. The scheme of the experiment is depicted in Fig. 6a while typical traces measured are shown in Fig. 6b. On approach, AFM tip does not feel any force; however, close to solid substrate one observes a jump-into contact. On retraction from the substrate, occasionally a single polymer molecule is picked up and stretched until the molecule detaches from the AFM tip or the substrate or when a carbon-carbon bond ruptures. The detachment or rupture point corresponds to the sudden return of positive force to zero as shown in Fig. 6b. The stretching part of the force profile is analyzed with the FJC model. This model predicts that the force is a universal function of the relative extension *x*/*L*, and thus the force profiles should collapse on a unique master curve. This master curve is shown in Fig. 6c. Thereby, the best-fit residuals are shown in Fig. 6d. One observes that the model provides an excellent description of the data. The fits yield an apparent Kuhn length ℓ= 0.6 ± 0.1 nm and a elasticity constant of *K* = 24 ± 3 nN. This Kuhn length corresponds to the length of a few monomeric units [1, 6, 30]. The elasticity constant is in very good agreement with the elasticity constant of poly(styrene) polymer molecules [30]. Inclusion of higher order corrections to the elasticity constant is possible [4]; however, given the accuracy of AFM force measurements, we suspect that the quadratic approximation in Eq. 3 remains valid.

The elasticity constant of poly(ethylene) polymer molecules is lower than that obtained from an extrapolation of the elasticity constants obtained from the computations to an infinite chain length. A possible explanation for the softer polymer elastic response is that there is a significant population of *gauche* structures in the polymer molecule. The *gauche* structures are softer than *trans* structures [2, 7, 32]. For example, while the elasticity constants of *trans*-butane are *K*_1_ = 47 nN and *K*_2_ = −101 nN, those of *gauche*-butane are *K*_1_ = 8 nN and *K*_2_ = −19 nN (Fig. S3).

At equilibrium, poly(ethylene) has a population of about 24% *gauche* structures, and 76% *trans*. These populations are estimated from Boltzmann distribution and the ground state energy difference between the two structures which is about 5 × 10^−21^ J calculated from MP2 (Fig. S4). The energy barrier between *gauche* to *trans* structural transition is about 20 × 10^−21^ J [33]. The area under force-extension profiles of poly(ethylene) molecule gives work that is input by the AFM cantilever (Fig. 6b). This work amounts to about 13(±4) × 10^−21^ J per monomer. The small energy barrier, together with the input AFM work suggests that *gauche* to *trans* structural transition may proceed in the AFM experiments by thermal activation. Using Bell model, one may estimate a maximum transition force of about 600 pN for such structural transition [34–36]. We observe that our force-extension profiles do not show a sign of structural transition akin to what has been observed for other systems which is in the form of force plateaus or kinks [27, 37–40]. Lack of such observation suggests that the transition occurs at much lower forces and at a rate that is obscured to the AFM. Moreover, our AFM measurements have a high force of more than 600 pN, where all monomers should be in the *trans* structure.

In contrast to AFM experiments, QC computations are in the gas phase and zero temperature. Environment effects generally reduce rupture forces of covalent bonds and rings [37–39], rupture force of physical bonds in supramoleucles [41], and *cis*-*trans* isomerization force of carbon-carbon double bonds [27, 40]. For example, while breakage of a covalent bond is expected to occur at around 5 nN in the gas phase [42], in the experimental condition it occurs at about 1–2 nN [37]. The impact of environment on facilitating structural transitions in molecules is consistent with computations by Politzer and co-workers [15]. We suspect that similar effects reduce the in-situ elastic response of poly(ethylene) as compared with the elastic response of an infinite alkane chain in the gas phase and zero temperature.

## Conclusions

Using the Hartree-Fock (HF) method of computational chemistry, it was previously shown that the energy-extension profiles of short alkane fragments are similar when normalized by their length. This result implied that the elasticity constants are intrinsic properties of these fragments. As a result, the elasticity constants of propane were used in interpretation of the elasticity of various polymer molecules. Using MP2, CCSD, and B3LYP methods, which are more accurate than HF due to inclusion of electron correlation energy, we show that the normalized energy profiles of alkane fragments are not the same. More importantly that the elasticity constant terms are not intrinsic properties of the fragments. The elasticity constants depend on length and are size extensive. They also depend on even and odd number of carbon atoms in the fragment. Both length and parity dependencies are found to diminish with the length of the fragments. It is then possible to extrapolate the elasticity constant terms to infinite chain length where we find a significantly stiffer elasticity constant than the elasticity constant of the actual polymer measured with AFM. The difference between the elastic properties obtained from high-level, gas-phase, zero-temperature calculations extrapolated to infinite chain length and the corresponding experimental data provides direct information about the contribution of the environment on the elastic response in a finite temperature.

## Electronic supplementary material

ESM 1 (DOCX 2067 kb)
