# Supplementary material for: Size extensivity of elastic properties of alkane fragments
Source: J Mol Model. 2018 Jan 8;24(1):36. doi: 10.1007/s00894-017-3572-9 (PMC5758687; doi:10.1007/s00894-017-3572-9)
Supplement: Supplementary file 1 — (DOCX 2067 kb) [file 894_2017_3572_MOESM1_ESM.docx]

Journal of Molecular Modeling

Supporting Information

**Size extensivity of elastic properties of alkane fragments**

Milad Radiom*^1,2^, Plinio Maroni^1^, Tomasz A. Wesolowski^3^

^1^Department of Inorganic and Analytical Chemistry, University of Geneva, Quai Ernest-Ansermet 30, 1205 Geneva, Switzerland

^2^Present address: School of Chemical Science and Engineering, KTH Royal Institute of Technology, Drottning Kristinas väg 51, Stockholm 10044, Sweden

^3^Department of Physical Chemistry, University of Geneva, Quai Ernest-Ansermet 30, 1205 Geneva, Switzerland

*Email: [miradi@kth.se](mailto:miradi@kth.se), Tel. +46 8 790 6642, ORCID: 0000-0002-6339-9288


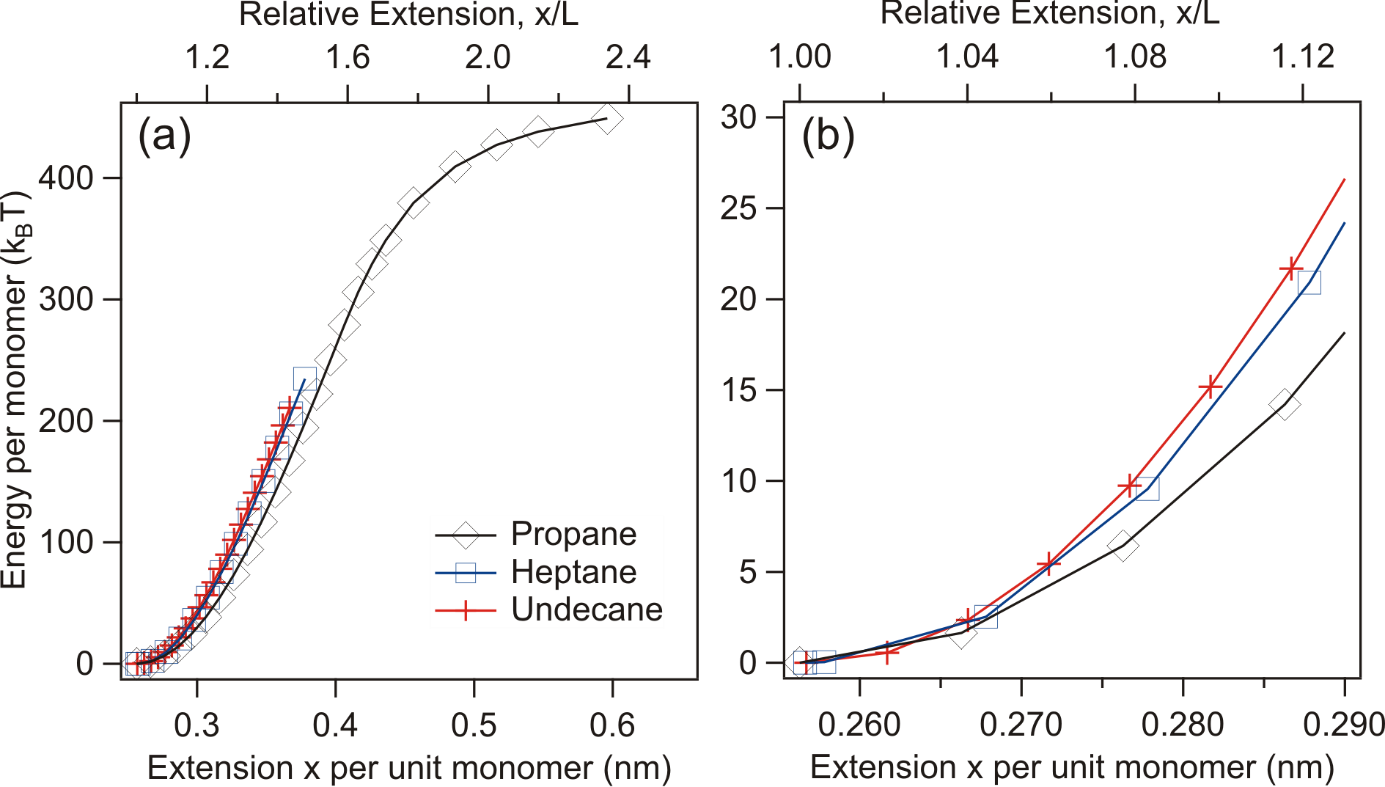


**Fig. S1** Energy per unit number of monomers for propane (number of monomers 1), heptane (number of monomers 3), and undecane (number of monomers 5) as a function of end-to-end extension per unit number of monomers (bottom x-axis), and as a function of relative extension (top x-axis). Computations are performed with Hartree-Fock (HF) and STO3G basis set method. (a) Computations over a long extension range. (b) Computations over an extension range that is relevant to AFM single molecule force spectroscopy. Even with HF method the energy profiles do not overlap in the extension range of AFM experiments.


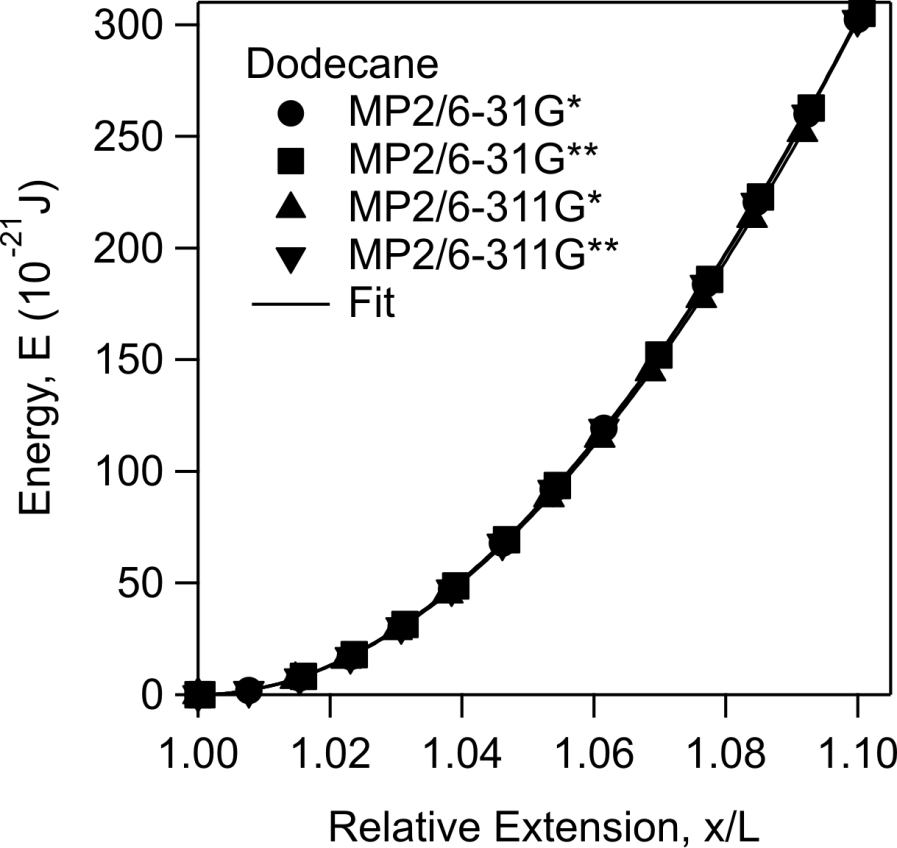


**Fig. S2** Energy as a function of relative extension profile of dodecane computed with MP2 theory and 6-31G*, 6-31G**, 6-311G* and 6-311G** basis sets together with the best fit of Eq. (1) using two elasticity constant terms. The resulting fit parameters are given in Table S1. No significant effect from basis set size is observed in these computations.


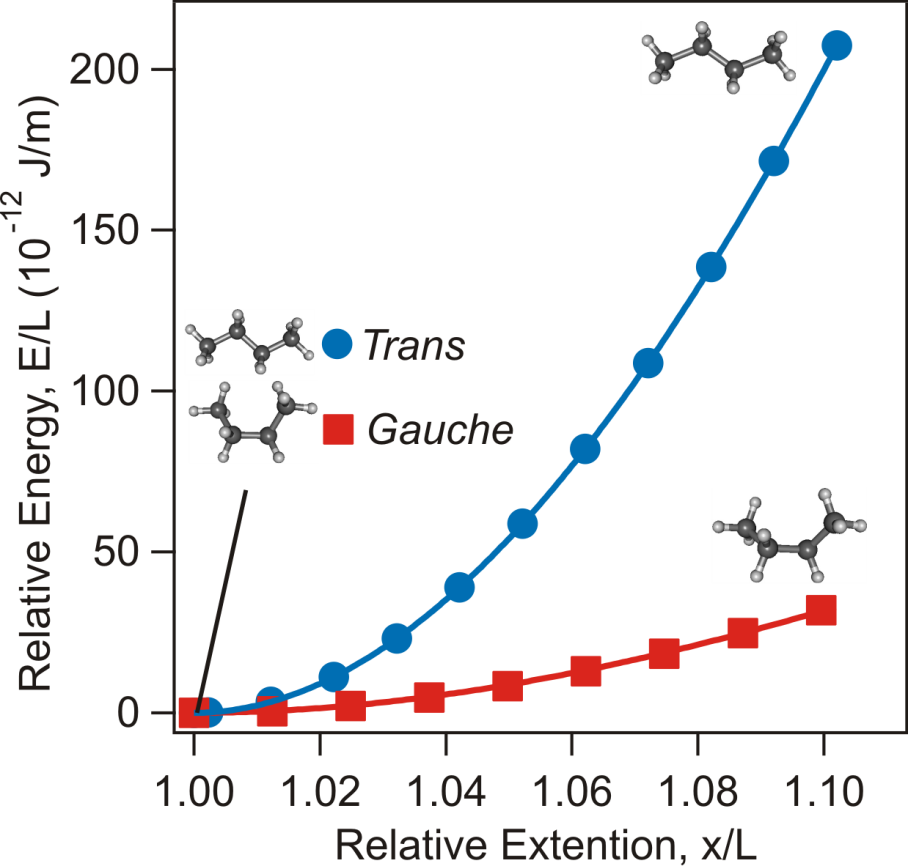


**Fig. S3** Relative energy as a function of relative extension profile of *trans*-butane and *gauche*-butane computed with MP2/6-31G* method together with the best fit of Eq. (1) using two elasticity constant terms. They are $K_{1}=47\pm0.1$nN and $K_{2}=-101\pm1$nN for *trans*-butane, and $K_{1}=8\pm0.1$nN and $K_{2}=-19\pm2$nN for *gauche*-butane. Structures at the ground state and extended state are shown. The *gauche* structure is much softer than the *trans* structure.


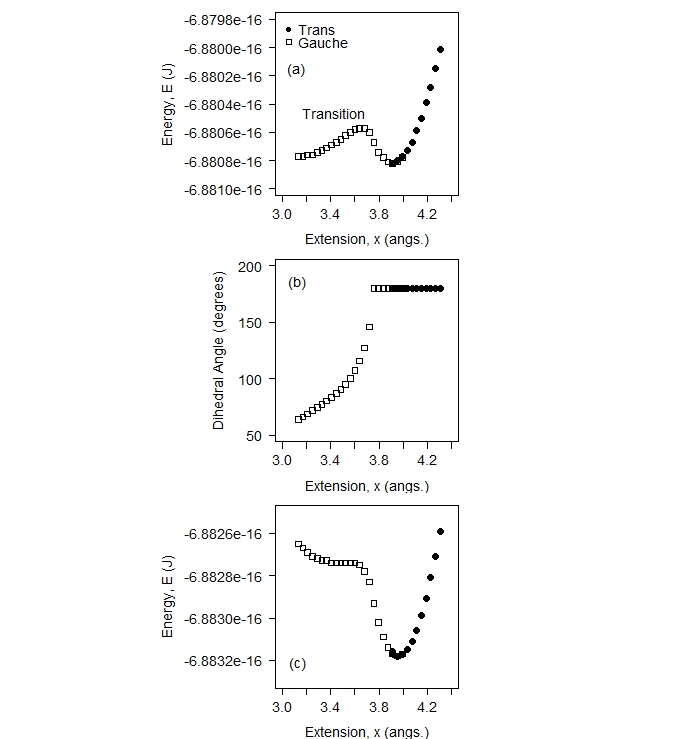


**Fig. S4** (a) Energy as a function of extension profile of *trans*- and *gauche*-butane computed with MP2/6-31G*. Transition point denotes transition from *gauche* structure to *trans* structure. The energy barrier is about $20\times10^{-21}$ J. The difference between energies of *gauche* and *trans* structures in their ground states is about $5\times10^{-21}$ J. (b) Dihedral angle‒extension profile. The angle varies from about 64^o^ to 180^o^ in *gauche*-butane. This angle remains constant 180^o^ in *trans*-butane. The transition point in (a) agrees with the point where complete rotation of the *gauche*-structure dihedral angle to 180^o^ occurs. (c) From Bell model, at a force of about 600 pN, the activation energy barrier for *gauche*-to-*trans* structural transition disappears. Since our AFM measurements of the force‒extension profile of poly(ethylene) has a high force of more than 600 pN, all monomers in the extended polymer are in *trans* structure.

**Table S1** The parameters resulting from best fits of energy profiles in Fig. S2 with Eq. (1). ± is 95% confidence interval in the fits.

|  | $K_{1} \left(\text{nN}\right)$ | $K_{2} \left(\text{nN}\right)$ |
| --- | --- | --- |
| 6-31G* | 48±1 | -68±3 |
| 6-31G** | 47±1 | -67±3 |
| 6-311G* | 47±1 | -65±3 |
| 6-311G** | 48±2 | -96±26 |

**Table S2** The parameters resulting from best fits of elasticity constant terms in Fig. 5 with Eq. (3).

|  | *a* | *b* | *c* |
| --- | --- | --- | --- |
| $K_{1} \left(\text{nN}\right)$ Odd | 5.221e-008 | -1.6895e-007 | 2.7985e-007 |
| $K_{1} \left(\text{nN}\right)$ Even | 5.1635e-008 | -7.5578e-008 | 2.2033e-007 |
| $K_{2} \left(\text{nN}\right)$ Odd | -3.1825e-008 | 5.4163e-007 | -1.4175e-006 |
| $K_{2} \left(\text{nN}\right)$ Even | -5.3332e-008 | -1.5089e-007 | -1.4336e-007 |
